# Development of Boron-Based Materials

**DOI:** 10.3390/ma18102247

**Published:** 2025-05-13

**Authors:** Nevill Gonzalez Szwacki

**Affiliations:** Faculty of Physics, University of Warsaw, Pasteura 5, 02093 Warsaw, Poland; gonz@fuw.edu.pl; Tel.: +48-22-55-32-797

The field of boron-based materials has undergone a significant transformation in recent decades, propelled by the unique chemistry of boron and its ability to form complex, multidimensional structures with exceptional physical, chemical, and electronic properties. With its electron deficiency and strong covalent bonding tendencies, boron forms a vast range of clusters, layers, and networks that do not find parallels among most other elements [1]. This Special Issue, entitled “Development of Boron-Based Materials”, brings together nine original research and review articles that collectively reflect the rapid expansion and diversification of the science of boron materials. These contributions represent a cross-section of the state-of-the-art approaches to synthesizing, characterizing, and theoretically modeling boron-rich compounds and composites with potential applications in electronics, energy storage, radiation shielding, catalysis, and biomedical science.

There are a range of motivations for investigating boron-based materials. For one, the light weight and abundance of boron make it attractive for sustainable applications. Furthermore, its structural versatility and ability to host metallic, semiconducting, insulating, and superconducting phases ensure a broad platform for technological innovations. From bulk ceramics and metallic borides to low-dimensional nanostructures and glasses, boron-containing systems demonstrate high hardness, chemical inertness, tunable band gaps, and exceptional thermal stability. These features, in turn, enable potential applications in armor systems, solid-state batteries, thermoelectrics, high-temperature electronics, and even quantum materials. Furthermore, current advances in computational modeling allow us to predict and screen novel boron configurations with a degree of precision that complements experimental synthesis and characterization.

Several contributions in this Special Issue focus on low-dimensional and nanoscale boron-based systems, which are at the forefront of condensed matter physics and material engineering. Arias-Camacho and Gonzalez Szwacki [2] present a computational study on the structural, electronic, magnetic, and transport properties of two-dimensional transition metal monoborides—specifically Cr, Fe, and Zr-based MBenes. These monolayer materials are theoretically stable in orthorhombic and hexagonal symmetries and display metallic behavior. Interestingly, significant magnetic moments in some of these configurations point to their potential utility as 2D magnets. Further extending the frontiers of two-dimensional boron science, Zhong et al. [3] investigate bilayer borophene, demonstrating a unique coexistence of superhard mechanical properties and tunable superconducting behavior. Their theoretical analysis shows that bilayer-$\delta_{6}$ borophene has a very high Young’s modulus and can reach a superconducting transition temperature up to 46 K under strain. These characteristics place it among the most promising 2D materials for mechanical and quantum applications. Yin et al. [4] contribute an experimental study on hydrogen boride (HB) sheets, focusing on the adsorption of atomic hydrogen. Using photoelectron spectroscopy, the authors identify chemical transformations at the boron surface, indicative of potential use in hydrogen storage and sensor technologies. These HB sheets are especially notable for their aqueous stability and functional surface chemistry. Perveen and Gonzalez Szwacki [5] explore the properties of borometallic molecular wheel clusters, where transition metal atoms are embedded in planar or drum-like boron ring structures. These clusters, predicted via first-principles calculations, are shown to be electronically stable and possess nontrivial magnetic properties. Their tunability suggests they may serve as fundamental units in future nanoscale spintronic devices.

Beyond low-dimensional systems, this Special Issue features studies that examine bulk boron-rich compounds for use in harsh environments. Iwan et al. [6] report the synthesis of a boron-rich boron carbide compound, B_4.55_C, via spark plasma sintering. Characterized by high hardness, thermal oxidation resistance, and neutron absorption capability, this material is suitable for high-temperature structural applications in aerospace and nuclear systems. In a more fundamental investigation, Werheit [7] explores the nature of phase transitions in boron carbide. His study identifies temperature- and pressure-induced transitions closely tied to subtle shifts in bonding and structural defects. These transformations are critical for understanding the anomalous electronic and thermal transport behaviors observed in boron-rich carbides. This Special Issue also explores functional boron-containing networks rooted in cluster and glass chemistry. Avdeeva et al. [8] provide a comprehensive review on the use of boron cluster anions and carboranes to generate advanced materials such as boron carbide, boron nitride, and metal borides. Their utility in protective coatings, composites, and neutron shielding materials is emphasized, showing the synthetic versatility of boron-rich precursors.

The protective capabilities of boron-based materials are well represented by the work of Avcıoğlu and Avcıoğlu [9], who evaluate transition metal borides as candidates for all-in-one radiation shielding. Using advanced simulation tools, they analyze gamma ray and neutron attenuation efficiencies. Compounds such as ReB_2_ and SmB_6_ are found to exceed the shielding performance of traditional lead-based materials, offering a more sustainable and less toxic alternative for shielding technologies in nuclear and aerospace contexts.

Finally, Kettlewell and Boyd [10] address the challenges of predicting structure–property relationships in oxyhalide borate glasses. These glasses, which operate in the complex “borate anomaly” regime, are modeled using a statistical design of mixtures methodology. Their study provides predictive frameworks for tuning degradation, ion release, and thermal stability—key features for biomedical and optoelectronic applications.

Altogether, the nine papers in this Special Issue offer a panoramic view of the current research landscape in boron-based materials. This collection demonstrates the diversity and depth of modern boron science, from the atomistic design of new 2D structures and bulk ceramics to the development of bioactive glasses and multifunctional composites. It highlights the synergies between theory and experiment, between chemistry and engineering, and between fundamental insights and applied objectives.
